# Recent Advances in Identifying Biomarkers and High-Affinity Aptamers for Gynecologic Cancers Diagnosis and Therapy

**DOI:** 10.1155/2019/5426974

**Published:** 2019-09-08

**Authors:** Xiaoqun Ma, Thangavel Lakshmipriya, Subash C. B. Gopinath

**Affiliations:** ^1^Deparment of Gynecology, Taian City Central Hospital, Taian, Shandong 271000, China; ^2^Institute of Nano Electronic Engineering, Universiti Malaysia Perlis, 01000 Kangar, Perlis, Malaysia; ^3^School of Bioprocess Engineering, Universiti Malaysia Perlis, 02600 Arau, Perlis, Malaysia

## Abstract

Cancer is the uncontrollable abnormal division of cell growth, caused due to the varied reasons. Cancer can be expressed in any part of the body, and it is one of the death-causing diseases. Human reproductive organs are commonly damaged by cancer. In particular, the women reproductive system is affected by various cancers including ovarian, cervical, endometrial, vaginal, fallopian tube, and vulvar cancers. Identifying these cancers at earlier stages prevents the damage to the organs. Aptamer is the potential probe that can identify these cancers. Aptamer is an artificial antibody selected from the randomized library of molecules and has a high binding affinity to the target biomarker. Targeting cancers in the reproductive organs using aptamers showed an excellent efficiency of detection compared to other probes. Different aptamers have been generated against the gynaecological cancer biomarkers, which include HE4, CA125, VEGF, OCCA (for ovarian cancer), EGFR, FGFR1, K-ras (for endometrial cancer), HPV E-16, HPV E-7, HPV E-6, tyrosine, and kinase (for cervical cancer), which help to identify the cancers in woman reproductive organs. In this overview, the biomarkers for gynecologic cancers and the relevant diagnosing systems generated using the specific aptamers are discussed. Furthermore, the therapeutic applications of aptamer with gynaecological cancers are narrated.

## 1. Introduction

**Figure 1:**
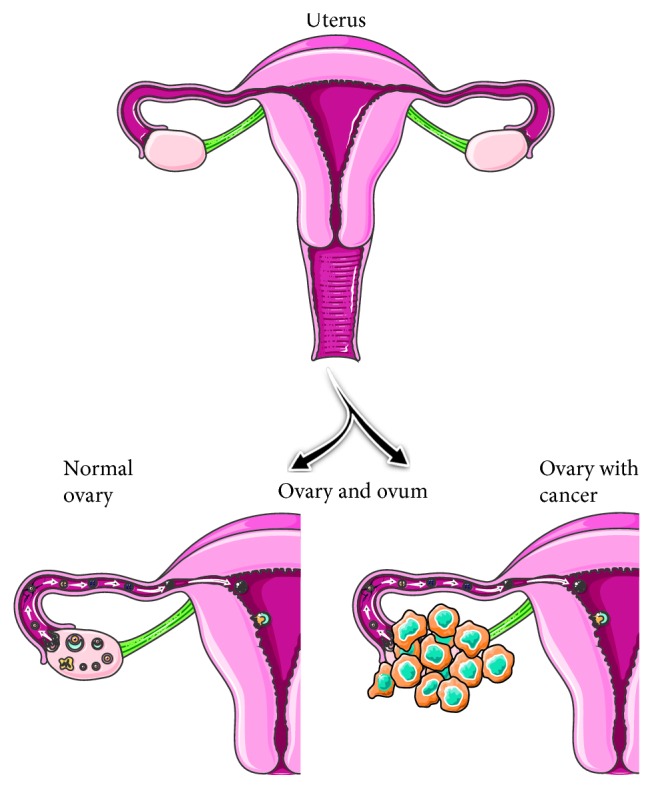
Representation on the uterus. The formation of cancer and normal uterus are shown.

Cancer is the abnormal cell growth in an uncontrollable way and a death-causing disease appearing in many parts of the body. More than 200 types of cancers have been identified. Cancers are caused by various reasons including genetic variation and exposure to chemicals [1, 2]. Reproductive organs of men and women are predominantly affected by cancers. In the case of men, the testicular, penile, and prostate are affected by cancers [3–5]. In women, all major parts in reproductive organs are affected by cancers which include endometrial cancer, ovarian cancer, cervical cancer, polycystic ovary syndrome, vaginal cancer, fallopian tube cancer, and vulvar cancer (Figure 1) [6–9]. According to American Cancer Society (ACS), the predominant and commonly recorded gynecologic cancers are cervical, uterine, ovarian, vaginal, and vulvar cancer. It is mandatory to identify these cancers at an earlier stage to protect the organs before getting damaged. Desiring or designing a suitable probe and biomarker helps to identify the cancers at an earlier stage. In general, DNA, RNA, antibody, protein, and aptamer are the probe to target the cancer cells for detection. Among them, aptamer is a high-affinity probe to the desired target molecule used to identify various diseases including cancer in an effective way.

**Figure 2:**
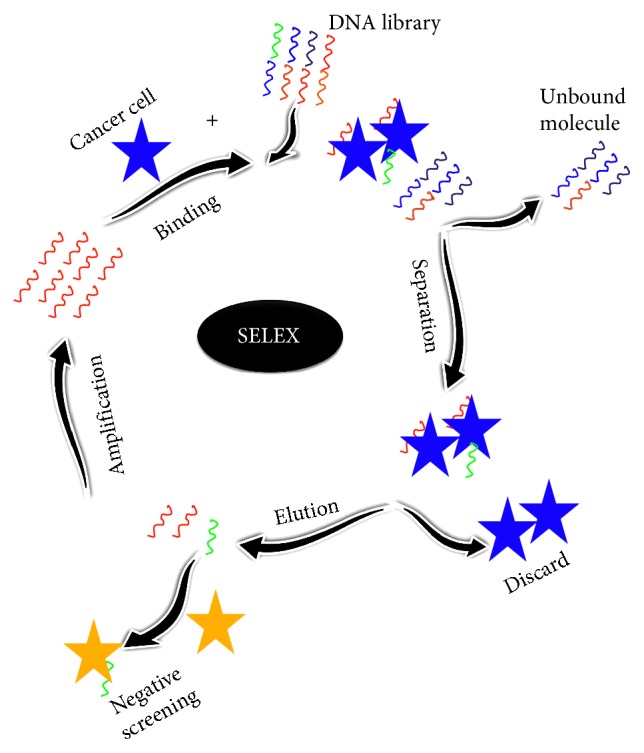
Schematic representation of cell-SELEX. It involves four steps including binding, separation, elution, and amplification. Negative screening is shown to remove the nonspecific binders.

The aptamer is an artificial antibody generated from the randomized library of molecules by the systematic evaluation of ligands by exponential enrichment (SELEX) method. SELEX involves four main steps, which include binding (the target with the selective molecule(s) from the randomized library), separation (the bound molecule(s) to the target from the unbound one), elution (the bound molecule(s) on the target), and amplification (the bound molecule(s)) [10–14]. The counterselection with other related molecules is drastically improving the SELEX with minimal cycles (Figure 2). Usually, to get the high-affinity aptamer, it is necessary to go for 5 to 10 SELEX cycles. After that, the selected molecules are cloned and sequenced to identify the specific aptamer. Through SELEX methods, DNA, RNA, XNA, and peptide aptamers are selected against a wide range of targets. They differ by the selection process, affinity, and secondary structure formation. DNA aptamer is used to generate directly using the synthesized DNA library by the SELEX method [12]. In the case of RNA aptamer generation, DNA pool needs to convert into RNA and this step has to follow in each selection cycle after amplifying the bound molecule from the target, by *in vitro* transcription [11]. Xeno nucleic acid (XNA) library is also desired in the aptamer studies by changing the sugar backbone of the oligonucleotides. It retains the genetic information and has a unique application in the field of xenobiology. Various DNAs and RNAs are selected against different targets ranging from a small molecule to the whole cell, such as intact viruses [12]. On the contrary, peptide aptamer selection has been performed using the peptide library with the artificial peptide loops based on the protein scaffold and yeast-two hybrid screening. It is predominantly involved in identifying the cellular protein binding to the peptide aptamer. Among these options, DNA and RNA aptamers have been generated widely, with the predominant number in DNA aptamers due to their stability in cellular milieu, whereas RNA aptamers need stable chemical modifications for *in vivo* applications. Furthermore, depending on the biomarker, the aptamer type will be desired [12]. The current overview is especially focused on the predominant nucleic acids (DNA and RNA) aptamers against the gynecologic cancers [15, 16]. The selected aptamers have positive features including high binding affinity, high selectivity, stability, and easier to be modified. Moreover, the nonimmunogenic nature of the aptamer is appealing, and it will not affect the physiology of the patients. The high-affinity aptamers with the target molecule are generally leading to the lower level of detection on sensors; it helps for the early diagnosis of gynaecological cancers. At the earlier stages of cancer progression, there is a lower level of target expression. At this situation, aptamers help to treat the patient and to avoid spreading further. With high-affinity aptamers, different clinical phase trials are in progress. Due to the high amenability of aptamers with the chemical modifications, stable aptamers have been generated and widely in use for clinical trials [12].

Sensitivity and specificity are the unavoidable criteria in any kind of detection strategy. Higher sensitivity is directly involved in the lower level of target detection. However, higher specificity is crucial for the detection for the desired target in the mixed biomolecules. Since blood or serum contains several proteins in addition to the specific biological element, there is a necessity to find a suitable detection probe with higher sensitivity and specificity. Aptamers are proved to have higher binding affinity and specificity to the targeted region of the protein, compared with other detection probes. Aptamers can be the efficient detection probe for gynaecological cancer targets as revealed.

**Figure 3:**
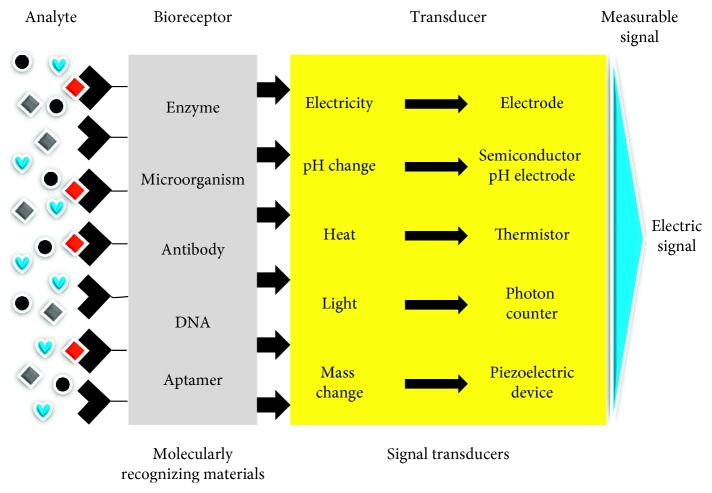
Biosensing strategy. A common sensing system involving probe and analyte along with the main parts, transducer, and signal output is shown.

**Figure 4:**
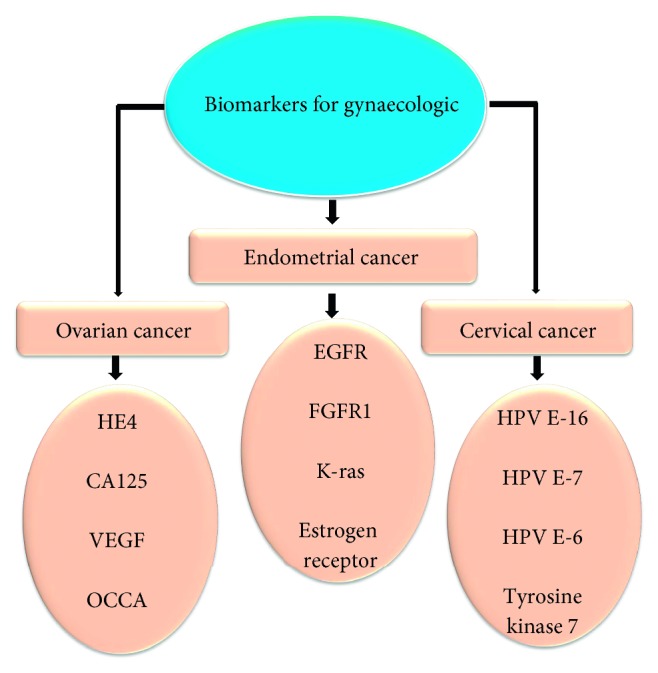
Biomarkers for gynecologic. Potential biomarkers for aptamer generation against ovarian, cervical, and endometrial cancers are listed.

Using aptamers as the potential analytical probe, several sensing strategies have been generated by following the general biosensor phenomena (Figure 3). Different strategies with aptasensing have been generated and show the advantages and disadvantages. Among the generated detections with label and label-free strategies, cell-based assays have a higher impact due to the *in vivo* nature. In addition, label-free methods are getting more priorities due to the lesser background signals. Apart from that, gold standard methods such as enzyme-linked immunosorbent assay give a strong validation to support other methods. Besides, nanoparticle-based colorimetry assays displaying the bare-eye detections can be performed without prior training (Table 1).  Figure 4 summarizes the biomarkers for generating aptamers against the gynaecological cancers. This overview discusses the suitable biomarkers for identifying the cancers in a female reproductive system using the aptamers.

## 2. Ovarian Cancer

Ovarian cancer is the fifth death-causing cancer in the United States, and every year around 22,000 cases have been recorded [26]. It is one of the gynecologic cancers, affecting all parts of the reproductive system including the uterus, ovary, and cervix. It has been recorded for the highest rate of death among other gynaecological cancers. It has four stages, and at stage I, it appears in the ovary or the fallopian tube. At stage II, it moves to the nearby tissues in the pelvis and uterus. In the next stage III, it spreads outside the pelvis and abdominal organs; at the last stage IV, it spreads to the distinct organ. Ovarian and other cancers are usually treated by chemotherapy and surgery. The success of the treatment is highly dependent on the stages of cancer. Early diagnose of the ovarian cancer is imperative to save the uterus and other organs. Finding the biomarkers from serum and transvaginal ultrasound are the common methods to detect ovarian cancer. However, these methods are not more specific to the concrete detection of ovarian cancer. Identifying the suitable biomarker and its probe are important in identifying ovarian cancer at an earlier stage.

### 2.1. Biomarkers for Ovarian Cancer

Different biomarkers are proposed to identify the ovarian cancer. Among those, CA 125 (cancer antigen 125) is the only FDA-approved biomarker for ovarian cancer. CA 125 is known as the transmembrane glycoprotein or mucin 16 with a higher molecular weight, and it is largely expressed in the epithelial ovarian cancer [27]. It is expressed on the cell surface and released into the blood serum. An elevated level with 50% of CA 125 expression is found at the early stage, and 80% of the elevated CA125 is at the advanced stage of ovarian cancer [28]. The monoclonal antibody CA125 is used to detect CA 125 antigen. After CA 125, human epididymis protein 4 (HE4) is known to be the efficient biomarker for ovarian cancer. Researchers found that 95% detection of ovarian cancer is by HE4 in the patient blood serum sample and HE4 is shown to have better detection than the observation with CA 125 [28]. The reference range for HE4 was fixed as 33.2 pmol/L, and HE4 is detected from the urine sample at all the stages of cancer with higher percentages [29, 30]. In addition, various malignancy levels of Matrilysin are also expressed and studies identified the relation of MMP-7 with ovarian cancer and its metastasis. The normal range of MMP-7 is 0–983 ng/mL. Wang et al. [31] have found the overexpression of MMP-7 with the malignant epithelium.

Glycodelin, a protein, is also found as one of the biomarkers for ovarian cancer; it is expressed in pregnancy deciduas, amniotic fluid, and endometrium [32]. It is especially secreted in various female-specific cancers including ovarian and breast colon and non-gender-specific cancers. Glycodelin is found in both normal and cancer-affected ovaries, but the level of glycodelin is drastically reduced in the women diagnosed with the premature ovarian failure. Compared with other biomarkers, glycodelin is found to be more specific for ovarian cancer [33]. Apart from that, mesothelin is the glycoprotein found on the cell surface, which was found to be overexpressed in 60% of ovarian cancer patients. Apolipoprotein A1 is the high-density lipoprotein in plasma, which is recognized to be decreased in the sera of the ovarian cancer patients [34]. There are different researches going on the relation of kallikreins (KLKs), vascular endothelial growth factor (VEGF), osteopontin, heat shock protein 10 (HSP 10), and prostasin with ovarian cancer [35].

### 2.2. Aptamer-Mediated Detection of Ovarian Cancer

Until now, the antibody is one of the desired probes to identify the biomarkers; at the same time, there are some limitations with antibodies, such as less stability, expensive, and having batch-to-batch variations. The aptamer is a substitute for the antibody, known as the artificial antibody, and has more positive characteristics, generated against a wide range of biomarkers including ovarian cancer. Through the efficient detection of these biomarkers using the specific probe, it is easier to identify ovarian cancer at an earlier stage. Since aptamer is one of the specified probes to most of the target molecules, it is possible to detect the cancer targets at a lower level. Various aptamers have been generated against various biomarkers to analyze and identify ovarian cancer. In general cell-SELEX (Figure 2), the intact cells have been used to generate the aptamer for the particular cell line. Van Simaeys et al. [36] have selected the high-affinity aptamer against two different cell lines, namely, TOV-21G and OCCA for ovarian cancer. The DNA aptamer for HE4 biomarker is generated by Eaton et al. [37] using the capillary electrophoresis SELEX; the dissociation constant of the aptamer for HE 4 is found to be within nanomolar range. These selected aptamers are used to detect the ovarian cancer.

**Figure 5:**
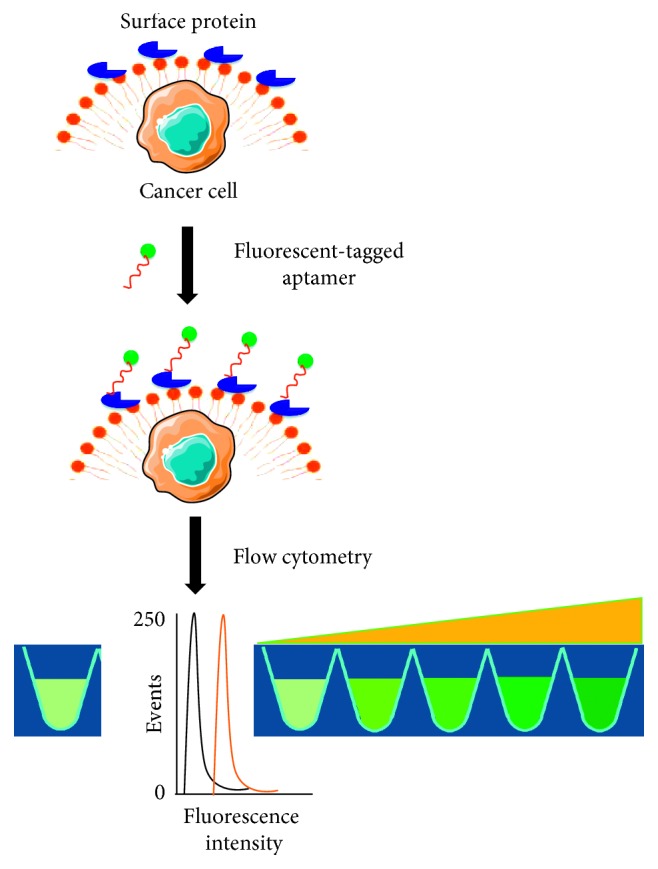
Detection of cancer by fluorescent-tagged aptamers. Upon binding of aptamer and surface protein, the intensity of fluorescence will be changed.

**Figure 6:**
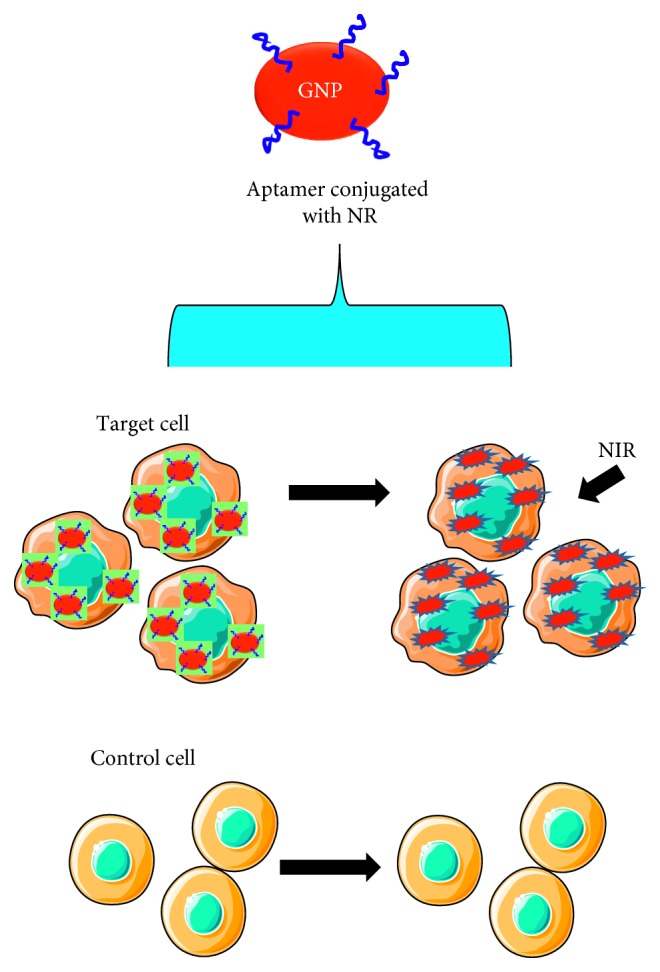
Schematic representation of photothermal therapy of cancer cells by aptamer-conjugated gold nanoparticles.

In another research, Chen et al. [17] used the aptamer to specifically detect CA 125 biomarker for recognizing ovarian cancer. The reference range for CA 125 is 38.3 U/mL, and there is a necessity to identify CA 125 lesser than the reference level [30]. Chen et al. [17] used the carboxyfluorescein- (FAM) labeled CA 125 aptamer and the fluorescence quenching method to identify CA 125. The detection limit is found as 0.05 U/mL (Figure 5). Since VEGF is related to many cancers, various aptamers are generated against VEGF [38, 39]. The normal range of VEGF is found to be lesser than 500 ng/mL [40]. The aptamer-based colorimetric assay is used to detect VEGF by Chang et al. [18]. In this colorimetric assay, the unmodified gold nanoparticle (GNP) has been used, in the presence of target aptamer. Then, the colour of free GNP changes to blue with a high salt concentration, for example, NaCl. In the absence of target, the aptamer binds on the surface of the GNP, and the colour of GNP remains in its original red colour even at a high salt concentration. The limit of detection of VEGF is to be 185 pM with this assay. Moreover, GNP-conjugated aptamer is also used for the photothermal therapy [41] (Figure 6).

## 3. Cervical Cancer

Cervical cancer is one of the predominant cancers appearing in the cervix, the lower portion of the uterus. It appears while the lined cells on the cervix undergo sudden changes. It is the third most common cancer in women and causes severe mortality and morbidity worldwide. It also has four main stages; at stage I, the cancer cell appears only in the cervix. At stage II, it started to move outside the cervix area, that is, the upper part of the vagina. At stage III, it spreads to the entire pelvis, and at stage IV, it spreads out of the pelvis. Cervical cancers are classified into three major types, namely, squamous cell carcinoma, adenocarcinoma, and adenosquamous carcinoma. Most of the cervical cancers are squamous cell carcinomas. In general, cancer can be cured when it has been found at the earlier stages. Cervical cancer has been used to be detected with CIN (cervical intraepithelial neoplasia) and Pap smear method [42]. But these systems have lack of reproducibility. Various biomarkers and probes are used to identify, screen, and treat cervical cancer.

### 3.1. Biomarkers for Cervical Cancer

Finding a suitable biomarker is mandatory to improve the detection. The best biomarker brings out the higher specificity and sensitivity and displays negative and positive predictive values. The associated human papillomavirus (HPV) is the well-identified biomarker for cervical cancer. HPV spreads through direct contact by the infected areas of mucous or skin membrane [43]. In general, it first affects the moist membranes of the body such as cervix, anus, throat, and mouth. The connection between HPV and cervical cancer is well studied [44]. In some cases, HPV leads to the origin of cervical cancer, and due to this reason, DNA of HPV has been used widely as the biomarker. Squamous cell carcinoma antigen (SCC-Ag) is also one of the established biomarkers for cervical cancer [45]. The increase in the level of SCC-Ag is the indication of cervical cancer.

Cytokeratin is found to be another biomarker for cervical cancer. An elevated level of cytokeratin has been detected in cervical cancer patients [46]. Matrix metalloproteinase (MMps) belongs to the protease family, and overexpression of MMPs-2 and -9 is found in the cervical cancer patient. Both MMP-2 and MMP-9 are mandatory matrix metalloproteinases playing a role in break down of the basement membrane, and it is necessary for the entry of cancer [47, 48]. Moreover, overexpression of minichromosome maintenance (MCM) gene is correlated with cervical cancer. This protein plays a necessary role in the progression of the cell cycle by the replication after DNA initiation and elongation. Das et al. [49] have found that MCM 2,4,6,7, and 10 are usually overexpressive in cervical cancer.

### 3.2. Aptamer-Mediated Nanodetection of Cervical Cancer

Different aptamers are generated against cervical cancer biomarkers for the purpose of detection and identification. Since aptamers are stable enough in the bloodstream upon modification, it is used to image cancer *in vivo*. Since HPV is an important biomarker for cervical cancer, aptamers have been generated against HPV. Toscano-Garibay et al. [23] synthesized an RNA aptamer against HPV-16 virus, and in particular, the selected aptamers are more specific to HPV E7 oncoprotein [23]. E6 and E7 are the HPV-16 oncogenes found in HPV-associated cancers. They have the capability to transform human tonsillar epithelial cells (HTECs). Aptamers are generated aiming to enter HPV-16 (E6/E7-HTECs). In general, cell-SELEX has been used to select the specific aptamer against cervical cancer. Various aptamers have also generated against HPV using the adherent cell lines by the whole-cell SELEX. This selected high-affinity aptamer is used in detection and imaging of cervical cancer. SERS-fluorescence-based assay with aptamers modifies silver-gold nanorod state and is used to detect cervical cancer. Another aptamer specific to the tyrosine kinase-7 human protein is generally expressed in cervical cancer [19]. HPV-E6-binding peptide aptamers are used to identify HPV E6 by oncoprotein [50]. It is found that the selected aptamer against HPV shows the apoptotic elimination of HPV-positive cancer cells. At the same time, it is not affected by the negative cells. This result concludes that HPV E-16 has the antiapoptotic activity in HPV-positive sample. Moreover, the nucleolin aptamer AS1411 is wrapped with carbon dots, and it is used to identify the cervical cancer by spectrofluorometry and the detection limit is 10 to 10^5^ cell/mL [50].

## 4. Endometrial Cancer

Endometrial cancer is also known as uterine cancer and originates by forming the cancer cells in the line of the endometrium. Worldwide, it is the 7^th^ most common cancer in women [51]. It also has four stages; at stage 1, it appears only in the upper part of the uterus. At stage II, it enters into the cervix; at stage III, it spreads into the vagina, nearby tissues, and lymph nodes. At the final stage IV, it moves to the intestine, bladder, and other parts of the body. Various factors cause endometrial cancer including metabolic syndrome, obesity, and consumption of medicines such as tamoxifen and estrogen [52, 53]. Identifying at the earlier stages of endometrial cancer helps to avoid damage of the uterus. Pelvic and transvaginal ultrasound is generally used to identify the endometrial cancer. Also, the endometrial biopsy is an accurate method to identify the endometrial cancer. All these methods are recognized to be expensive and complicated. If the detection method is designed for the biological samples such as urine and blood serum, it will be useful to identify the cancer. Sometimes, the whole blood test is taken to count the blood cells. Due to the overbleeding caused by endometrial cancer, the red blood cell count will be lower in the patient. In this way, they can assume the patient condition but cannot say accurately. Suitable biomarkers with the right probe are necessary to identify endometrial cancer at earlier stages at the lower abundance. All the cancer progressions are varying with the biomarker expression levels. These variations are either upregulated or downregulated from their normal level. With the downregulation, there is an expectation of associated target at a lower level.

### 4.1. Biomarkers for Endometrial Cancer

Identifying a suitable biomarker helps to improve the diagnosis method. The progesterone receptor, estrogen receptor, mutated PTEN, K-ras, p53, oncogenes, and HER2/neu mutation have been found as the potential biomarkers for endometrial cancer. It is found that there is a positive association between the positive estrogen and progesterone with endometrial cancer [54]. Also, PTEN tumor-suppressor gene mutation is found in endometrial cancer [55]. At the same time, it is found that K-ras gene is overexpressed but not mutated in endometrial cancer [56]. In another research, Alkushi et al. have found the presence of p53 in endometrial cancer [57]. In addition, oncogene, fibroblast growth factor receptor 2 (FGFR2), P13KCA, and epidermal growth factor receptor (EGFR) are also playing a necessary role in endometrial cancer. FGFR2, a tyrosine kinase receptor, is mainly involved in the process of tissue homeostasis, embryogenesis, and cell proliferation. Mutations in FGFR2 are found (10–12%) in the endometrial cancers [58]. A significant amount of EGFR expression is found in endometrial cancer. Scambia et al. [59] have stated that out of 26 endometrial cancer patients, 13 are expressed as the significant level of EGFR.

### 4.2. Aptamer-Mediated Detection of Endometrial Cancer

The overexpression of EGFR is found in several cancers including endometrial cancer. Different DNA and RNA aptamers are generated against EGFR. Deng et al. [60] synthesized DNA aptamer against EGFR/HER1/c-ErbB1, and the dissociation constant of the selected aptamer is found to be ∼7.3 nM. Also, aptamers are generated against the fibroblast growth factor receptor type-1 (FGFR1) for anticancer hyperthermia therapy. It is found that the aptamers selectively targeted and destroyed the cancer cells [61]. Jeong et al. [62] have generated the aptamer specific for the mutant K-ras protein. The selected aptamer's binding affinity with K-ras is confirmed by surface plasmon spectroscopy and real-time polymerase chain reaction. The dissociation constant is noted as ∼4 nM [62]. Wang et al. [63] also generated the different DNA aptamers for RAS protein and showed the dissociation constant of 15.3 nM. DNA aptamer for estrogen receptor is synthesized by Sett et al. [64]. In addition, *in silico* aptamer selection for estrogen receptor is demonstrated using RNA analogs of estrogen response elements from a human.

## 5. Conclusion

Herein, overviewed the advancements in identifying biomarkers and high-affinity aptamers for gynecologic cancers diagnosis and therapy, which includes cervical, uterus and endometrial cancers. Special focus has been given to aptamers selected against a wide range of biomarkers for gynecologic cancers. For the past few decades, aptamers are replacing other probes due to their uniqueness such as amenability to the chemical modifications to yield the stability under the stringent conditions. The detection of HPV with aptamer shows the higher performance than the detection by using the commercial kits because methods with aptamers are more sensitive and selective to the target. In addition, due to the higher affinity of the aptamer, there is a possibility of detecting cancers at the earlier stages. A brief outline is given here on the aptasensing strategy which creates new paths for the efficient detection of gynaecological cancers. In addition, aptamers aid to create the ways for the therapeutic approaches. Further expansion using the currently available aptamers and the aptamer-based sensing methods will open a new avenue. Critical analysis and chemical stabilization on aptamers and proper filling of gap between aptamer generation and sensing methods will make a drastic therapeutic movement.

## Figures and Tables

**Table 1:** Summary on biomarkers and aptamers with detection strategies.

Gynaecological cancer	Biomarker	Aptamer type	Diagnosing method	Limit of detection	Advantage/disadvantage	Reference
Ovarian cancer	CA125	DNA	Fluorescence quenching	0.05 U/mL	Sensitive, need background optimization	[17]
Ovarian cancer	VEGF	DNA	Colorimetric assay	185 pM	Less sensitive, visual detection	[18]
Cervical cancer	PTK-7	DNA	RAMAN scattering	—	Need training personnel	[19]
Cervical cancer	PTK-7	DNA	Cytosensor	10-10^6^ cells/mL	*In vivo* reflects real condition	[20]
Endometrial cancer	EGFR	RNA	Electrical	—	Sensitive, label free	[21]
Endometrial cancer	EGFR	DNA	Electro chemical	50 pg/mL	Sensitive, consume more sample volume	[22]
Cervical cancer	HPV-16 E-7	RNA	Radio isotope	Kd 1.9 *µ*M	Sensitive, aptamer needs stabilization	[23]
Cervical cancer	HPV-16	DNA	ELISA	—	Gold standard, less sensitive	[24]
Ovarian cancer	CA 125	DNA	FET sensor	5.0 × 10^−9^ U/mL	Sensitive, label free	[25]
